# Guillain–Barré Syndrome Emerging From Syringomyelia Management: A Case Report

**DOI:** 10.1155/carm/8542341

**Published:** 2026-06-24

**Authors:** Abbigail Niewchas, Brandon Baccari, Christopher Stewart, Suporn Sukpraprut-Braaten, Donald G. Smith

**Affiliations:** ^1^ College of Osteopathic Medicine, Kansas City University, Joplin, Missouri, USA, kcumb.edu; ^2^ Department of Family Medicine, Reid Health, Richmond, Indiana, USA; ^3^ Graduate Medical Education Consortium, Kansas City University, Kansas City, Missouri, USA, kcumb.edu

## Abstract

The coexistence of Guillain–Barré syndrome with a spinal cord syrinx is extremely rare. This report presents the emergency admission of a 57‐year‐old female presenting with an abrupt and progressive loss of motor function and abnormal sensation in her lower extremities. MRI of the cervical spine revealed a syrinx extending from C2 to C7. Neurology referred the case to neurosurgery, who assessed the patient and recommended nonoperative and supportive treatment given the size of the syrinx and the patient’s status as a poor surgical candidate. She was subsequently discharged to a local extended care facility (ECF) with a diagnosis of syringomyelia. Despite physical therapy and rehabilitation, her symptoms progressed to include loss of motor function in bilateral lower extremities, worsening paresthesia in bilateral upper and lower extremities, and progressive dyspnea. Nerve conduction studies performed during neurologic evaluation at the ECF demonstrated a sensorimotor demyelinating polyneuropathy, and she was urgently transferred to the emergency department, where Guillain–Barré syndrome was confirmed. The patient developed respiratory failure requiring mechanical ventilation and was treated with intravenous immunoglobulin. Although she experienced transient stabilization, her clinical course was complicated by progressive functional decline. This case highlights how a cervical syrinx may obscure the initial presentation of Guillain–Barré syndrome, contributing to delayed treatment, and demonstrates the importance of maintaining a broad differential diagnosis in patients with atypical or overlapping neurologic findings.

## 1. Introduction

Guillain–Barré Syndrome (GBS) is an acute, immune‐mediated polyneuropathy that remains the most common cause of rapidly progressive paralysis in developed countries [1]. In the United States alone, an estimated 3000 to 6000 new cases of GBS are reported annually [2]. The classic presentation is described as an acute onset, bilateral paresthesia followed by progressive extremity weakness from distal to proximal, but atypical presentations are well documented, and presenting symptoms can vary significantly, especially in its early stages [3]. In addition to the inherent difficulty of diagnosing GBS at initial presentation, this case was further complicated by a neurologic finding that obscured the clinical picture: the presence of a cervical syrinx. Syringomyelia is characterized by abnormal cerebrospinal fluid circulation, leading to the formation of fluid‐filled cavities (syrinx) within the spinal cord parenchyma or central canal [4]. The estimated prevalence of syringomyelia varies widely across geographic and demographic groups but has been reported as 8.4 per 100,000 people in Western countries [4]. Syrinx can be classified according to its communication or lack of communication with the fourth ventricle of the brain. This classification, combined with the location and size, has implications for both the presenting symptoms and treatment indications for neurosurgery [5, 6].

GBS with concurrent syringomyelia has been reported only twice in the literature [7, 8]. In this report, we present a rare case of GBS initially masked by a cervical syrinx, underscoring the importance of thorough neurological examination and comprehensive diagnostic testing even when preliminary findings are inconclusive. This case offers novel insight through unique patient demographics, an atypical presentation, and a clinical course not previously reported, thereby expanding the current understanding of the diagnostic challenges associated with concurrent GBS and syringomyelia.

## 2. Case Presentation

A 57‐year‐old White female presented to the Emergency Department with a primary concern of bilateral tingling and weakness in her lower extremities for the past 24 h. She reported that her symptoms had progressed rapidly that morning and that her weakness nearly caused her to fall over while standing in her kitchen. At that point, she contacted emergency services and was promptly transported to the Emergency Department. Past medical history included hypothyroidism, mixed hyperlipidemia, gastroesophageal reflux disease without esophagitis, mixed irritable bowel syndrome, generalized anxiety disorder, and recurrent major depressive disorder. Past surgical history was notable for cholecystectomy and tonsillectomy in the remote past. Her medications at the time of presentation included escitalopram 20 mg daily, levothyroxine 50 mcg daily, pravastatin 80 mg daily, celecoxib 200 mg daily, and alprazolam 1 mg three times daily. The only medication allergy was to sertraline. Her family history was negative for neurological diseases. Social history revealed smoking cessation more than 2 decades before admission, and she did not use alcohol or illicit drugs. There was no history of trauma before symptom onset.

Vital signs on admission revealed a temperature of 98.3°F, blood pressure of 151/89 mmHg, pulse of 106 beats per minute, respiration of 18 breaths per minute, and oxygen saturation of 94%. Her weight was 348 pounds, with a Body Mass Index of 59 kg/m^2^. On examination, the patient was calm, cooperative, and in no acute distress. She was oriented with normal cognition and communication. On physical examination, strength was found to be diminished in the lower extremities, graded as 4/5, which impaired her ability to stand upright or ambulate without the use of her walker. Patellar reflexes were 1+ bilaterally, and Achilles reflexes were 0 bilaterally. Upper extremity strength was preserved at 5/5 bilaterally, with biceps and triceps deep tendon reflexes graded as 2+ bilaterally. She reported normal sensation in her upper extremities; however, she had notable hyperesthesia in the distal lower extremities bilaterally. Cranial nerve testing, coordination, and fine motor testing of the upper extremities were normal. Gait evaluation revealed that the patient was able to ambulate only a few steps with the assistance of a walker before becoming easily fatigued and requesting to stop. Further history revealed that she had been largely sedentary for several weeks preceding symptom onset, which was initially considered a potential contributing factor to her weakness. Urinalysis did not reveal any evidence of infection, and the respiratory panel, including SARS‐COV2 PCR, was negative. Laboratory studies revealed an elevated blood urea nitrogen, glucose, C‐reactive protein, and erythrocyte sedimentation rate. A summary of initial laboratory results can be found in Table 1.

**TABLE 1:** Patient’s emergency room laboratory results at the initial visit.

Lab		Value		Normal range
White blood cell	WBC	9.4	× 10^9^/L	4.5 to 11.0 × 10^9^/L
Hemoglobin	HGB	13.9	g/dL	12.1 to 15.1 g/dL
Hematocrit	HCT	42.9	%	36% to 48%
Platelet count	PLTS	294,000	platelets/mcL	150,000 to 400,000 platelets/mcL
Sodium	Na	139	mEq/Ls	135 to 145 mEq/L
Potassium	K	4.1	mmol/L	3.6 to 5.2 mmol/L
Chloride	Cl	104	mEq/L	96 and 106 mEq/L
Blood urea nitrogen	BUN	11	mmol/L	2.1 to 8.5 mmol/L
Creatinine	Crea	0.8	mg/dL	0.5–1.0 mg/dL
Glucose	Glu	111	mg/dL	70 to 100 mg/dL
Alanine transaminase	ALT	32	U/L	4 to 36 U/L
Aspartate aminotransferase	AST	21	U/L	8 to 33 U/L
C‐reactive protein	CRP	21	mg/dL	3–10 mg/L
Erythrocyte sedimentation rate	ESR	46	mm/hour	< 30 mm/hour
Troponin I^∗^	cTnI	< 0.012	ng/mL	0.012–0.120 ng/mL
B‐type natriuretic peptide	BNP	35.0	pg/mL	< 100 pg/mL

^∗^Including repeat measurement at 3 h postpresentation.

Initial radiologic studies included CT of the head, cervical spine, and lumbar spine and did not reveal any findings that could explain the patient’s symptoms on presentation. An incidental left thyroid nodule, mild degenerative changes in the lumbar spine, and possible bilateral upper lung infiltrates were reported. A subsequent chest radiograph confirmed bilateral infiltrates; however, a comparison with prior imaging suggested that these findings did not represent an acute cardiopulmonary process, according to the radiology report. MRI of the brain was negative for intracranial pathology. MRI of the cervical and thoracic spine was notable for a cervical spinal cord syrinx extending from C2 to C7, with the greatest diameter from C4 to C6 (Figure 1).

**FIGURE 1:**
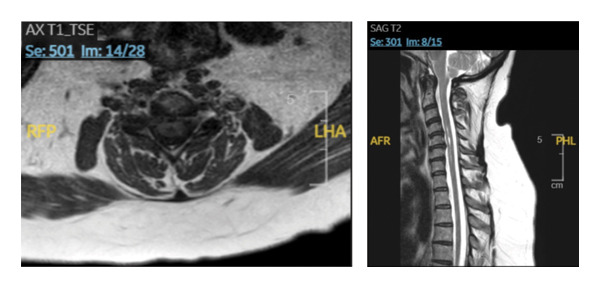
Magnetic resonance imaging of the cervical spine revealed a spinal cord syrinx extending from C2 to C7. (A) T1 sequence, axial view; (B) T2 sequence, sagittal view.

The cervical syrinx, consistent with syringomyelia, was initially considered the underlying cause of the patient’s symptoms and abnormal neurologic findings. However, the rapid progression of symptoms over 24 h was atypical for syringomyelia and raised concern for an acute neuromuscular process. In‐house neurology service was therefore contacted and recommended consultation with a neurosurgery team at a tertiary center in the nearest large city. Neurosurgical consultation was performed to review the patient’s records and radiologic studies. This team concluded that surgical intervention for the patient’s syringomyelia was not indicated, considered her comorbidities to be potential contributors to her symptoms, and declined transfer. Due to her inability to ambulate without a walker, the patient was transferred to a local extended care facility (ECF) for rehabilitation with a scheduled follow‐up with the local neurologist for nerve conduction testing.

Despite undergoing physical therapy at the ECF, the patient’s symptoms did not improve. She reported slowly progressive and worsening numbness in the lower extremities. Approximately two weeks after her initial emergency department visit, neurology was able to visit her in the ECF. At this time, she was determined to have a complete loss of strength and movement in the lower extremities (0/5 bilaterally) and profound weakness in the upper extremities (2/5 bilaterally), accompanied by progressive dyspnea. Nerve conduction velocities and electromyogram (EMG) were conducted on site, with the findings concerning for a sensorimotor demyelinating polyneuropathy. There were no reported symptoms of dysphagia, dysarthria, or other bulbar involvement, so the patient’s respiratory symptoms were attributed to progressive respiratory muscle weakness in the setting of advancing neuromuscular disease. She was immediately transported to the hospital. On arrival, CSF analysis was significant for elevated protein (120 mg/dL), very mildly elevated white blood cell count (7 cells/µL), and the presence of P23 Ab IgG. Repeat nerve conduction studies demonstrated absent motor responses in the peroneal and tibial nerves, markedly reduced CMAP amplitudes in the median and ulnar motor nerves, prolonged distal motor latencies, and slowed conduction velocities in the bilateral median nerves. Sensory nerve responses in both the upper and lower extremities were either absent or markedly reduced. These findings were consistent with a length‐dependent sensorimotor demyelinating polyneuropathy. Combined with the patient’s symptoms on prior admission and re‐presentation, a formal diagnosis of GBS was made.

The patient’s respiratory status continued to deteriorate, and she was transferred to the intensive care unit for intubation and initiation of intravenous gamma globulin (IVIG) treatment at a standard dose of 0.4 g/kg/day for 5 days. Plasma exchange was considered as an alternative therapy; however, IVIG was selected in accordance with institutional practice and clinical considerations. After the recovery of normal respiration and stabilization of her condition, she was again transferred to the ECF for rehabilitation. Despite intensive rehabilitation, the resolution of her symptoms was unsuccessful, and the patient effectively became bedridden. Unfortunately, the patient later developed a series of infections and sadly passed away from complications surrounding her multiple comorbid conditions.

## 3. Discussion

This case highlights the diagnostic challenge posed by overlapping neurologic pathologies, in which the presence of a structural abnormality obscured the recognition of an evolving immune‐mediated process. At initial presentation, the patient demonstrated rapidly progressive lower extremity weakness, hyporeflexia, and sensory abnormalities. GBS was considered as a possible diagnosis early in the disease course, and the discovery of bilateral pulmonary infiltrates on initial imaging raised consideration of a preceding infectious trigger. However, comparison with prior imaging suggested that these findings did not represent an acute pulmonary process, and respiratory infectious testing at presentation was negative. Further diagnostic imaging revealed a cervical syrinx, providing a plausible structural explanation for her symptoms [4]. Combined with the patient’s morbid obesity, prolonged sedentary status, and multiple comorbidities, deconditioning and functional decline were considered contributing factors to her gait impairment and weakness. In retrospect, the rapid progression of symptoms over 24 h and the peripheral pattern of neurologic findings were less characteristic of syringomyelia and more consistent with an evolving neuromuscular disorder such as GBS.

On re‐presentation, the patient exhibited marked progression of neurologic deficits, including complete loss of lower extremity strength, worsening upper extremity weakness, and respiratory compromise. Electrophysiological studies and cerebrospinal fluid analysis ultimately confirmed the diagnosis of GBS in the setting of coexisting syringomyelia [9, 10], and the patient was promptly initiated on first‐line immunotherapy [3] and respiratory support. IVIG was selected in accordance with institutional practice patterns and evidence demonstrating comparable efficacy between IVIG and plasma exchange in the treatment of GBS [11]. The patient’s later respiratory deterioration was felt to be more consistent with progressive neuromuscular respiratory weakness than an acute pulmonary process, as the infiltrates identified on initial imaging were interpreted as chronic and noninfectious at the time of presentation.

The coexistence of syringomyelia with GBS has been reported only rarely in the literature [7, 8], and this report further supports the principle that the presence of one neurologic diagnosis does not exclude the possibility of another. Diagnostic uncertainty was further compounded by the presence of a cervical syrinx, multiple comorbidities, and healthcare limitations associated with the early stages of the COVID‐19 pandemic in a nontertiary care setting. Following neurology consultation, the diagnostic focus shifted toward neurosurgical evaluation of the syrinx to determine whether the structural abnormality could account for the patient’s presenting symptoms and whether surgical intervention was indicated. Upon reflection on the diagnostic pathway, this case likely illustrates elements of anchoring bias and premature diagnostic closure. The identification of a plausible structural lesion narrowed the diagnostic focus despite clinical features increasingly suggestive of a concurrent peripheral neuropathic process. It also underscores the importance of comprehensive neurologic reassessment when the clinical course evolves beyond what would be expected from the presumed etiology. While early neurological consultation has been associated with improved diagnostic accuracy and clinical outcomes in patients with GBS [12], delayed recognition remains common and may still occur in patients with atypical presentations, significant comorbidities, or competing neurologic diagnoses, as demonstrated in this case [13, 14]. Additionally, diagnostic studies in GBS may remain normal or nonspecific when obtained early in disease progression [15–18], further complicating recognition during initial presentation. Finally, it is possible that earlier recognition and initiation of treatment may have improved the outcome, as timely treatment has been associated with improved functional recovery and reduced morbidity [19, 20].

The patient initially demonstrated improvement in the intensive care unit and was successfully extubated. However, her subsequent clinical course and eventual death were likely influenced by delayed diagnosis and initiation of immunotherapy [20, 21], severe comorbidities including morbid obesity and major depressive disorder, prolonged immobility, and infectious complications following return to the ECF [19, 22]. Ultimately, mortality in this case was attributed to complications related to prolonged hospitalization and comorbid conditions rather than GBS alone.

## 4. Conclusion

With this case report, we highlight a rare instance in which the presence of a spinal cord syrinx in a patient presenting with upper and lower extremity paresthesia, and weakness obscured the diagnosis of GBS. In patients presenting with atypical neurologic findings, clinicians should maintain a broad differential diagnosis and prioritize comprehensive neurologic evaluation, even when a structural abnormality such as a spinal cord syrinx is identified. In addition to a thorough neurologic examination, nerve conduction studies, electromyography, and cerebrospinal fluid analysis should be considered early on to exclude GBS and prevent delays in appropriate treatment.

## Author Contributions

Brandon Baccari, Christopher Stewart, Abbigail Niewchas, Donald G. Smith, and Suporn Sukpraprut‐Braaten provided substantial contributions to conception and design, acquisition of data, or analysis and interpretation of data; Abbigail Niewchas, Brandon Baccari, Christopher Stewart, Donald G. Smith, and Suporn Sukpraprut‐Braaten drafted the article or revised it critically for important intellectual content; Brandon Baccari, Donald G. Smith, and Suporn Sukpraprut‐Braaten gave final approval of the version of the article to be published.

## Funding

No funding was received for this research.

## Disclosure

All authors agree to be accountable for all aspects of the work in ensuring that questions related to the accuracy or integrity of any part of the work are appropriately investigated and resolved.

## Consent

The next of kin of the patient in this study provided verbal and written informed consent for the patient’s case and images to be used.

## Conflicts of Interest

The authors declare no conflicts of interest.

## Data Availability

Data sharing is not applicable to this article as no datasets were generated or analyzed during the current study.
